# Cure of Disseminated Human Lymphoma with [^225^Ac]Ac-Ofatumumab in a Preclinical Model

**DOI:** 10.2967/jnumed.122.265167

**Published:** 2023-06

**Authors:** Mark S. Longtine, Kyuhwan Shim, Mark J. Hoegger, Nadia Benabdallah, Diane S. Abou, Daniel L.J. Thorek, Richard L. Wahl

**Affiliations:** 1 Mallinckrodt Institute of Radiology, Washington University School of Medicine, St. Louis, Missouri;; 2 Department of Biomedical Engineering, Washington University in St. Louis, St. Louis, Missouri; and; 3 Department of Radiation Oncology, Washington University School of Medicine, St. Louis, Missouri

**Keywords:** CD20, lymphoma, radioimmunotherapy, ^225^Ac

## Abstract

Immunotherapies that target the CD20 protein expressed on most non-Hodgkin lymphoma cells have improved clinical outcomes, but relapse is common. We prepared ^225^Ac-labeled anti-CD20 ofatumumab and evaluated its in vitro characteristics and therapeutic efficacy in a murine model of disseminated human lymphoma. **Methods:** ^225^Ac was chelated by DOTA-ofatumumab, and radiochemical yield, purity, immunoreactivity, stability, and chelate number were determined. In vitro cell killing of CD20-positive, human B-cell lymphoma Raji-Luc cells was assayed. Biodistribution was determined as percentage injected activity per gram (%IA/g) in mice with subcutaneous Raji-cell tumors (*n* = 4). [^225^Ac]Ac-ofatumumab biodistribution in C57BL/6N mice was performed to estimate projected human dosimetry. Therapeutic efficacy was tested in mice with systemically disseminated Raji-Luc cells, tracking survival, bioluminescence, and animal weight for a targeted 200 d, with single-dose therapy initiated 8, 12, or 16 d after cell injection, comparing no treatment, ofatumumab, and low (3.7 kBq/mouse) and high (9.25 kBq/mouse) doses of [^225^Ac]Ac-IgG and [^225^Ac]Ac-ofatumumab (*n* = 8–10/cohort). **Results:** Radiochemical yield and purity were 32% ± 9% and more than 95%, respectively. Specific activity was more than 5 MBq/mg. Immunoreactivity was preserved, and more than 90% of the ^225^Ac remained chelated after 10 d in serum. Raji-Luc cell killing in vitro was significant, specific, and dose-dependent. In tumor-bearing mice, [^225^Ac]Ac-ofatumumab displayed low liver (7 %IA/g) and high tumor (28 %IA/g) uptake. Dosimetry estimates indicated that bone marrow is likely the dose-limiting organ. When therapy was initiated 8 d after cell injection, untreated mice and mice treated with cold ofatumumab or low- or high-dose [^225^Ac]Ac-IgG showed indistinguishable median survivals of 20–24 d, with extensive cancer-cell burden before death. Low- and high-dose [^225^Ac]Ac-ofatumumab profoundly (*P* < 0.05) extended median survival to 190 d and more than 200 d (median not determinable), with 5 and 9 of 10 mice, respectively, surviving at study termination with no detectable cancer cells. Surviving mice treated with high-dose [^225^Ac]Ac-ofatumumab showed reduced weight gain versus naïve mice. When therapy was initiated 12 d, but not 16 d, after cell injection, high-dose [^225^Ac]Ac-ofatumumab significantly extended median survival to 40 d but was not curative. **Conclusion:** In an aggressive disseminated tumor model, [^225^Ac]Ac-ofatumumab was effective at cancer-cell killing and curative when administered 8 d after cell injection. [^225^Ac]Ac-ofatumumab has substantial potential for clinical translation as a next-generation therapeutic for treatment of patients with non-Hodgkin lymphoma.

## Visual Abstract

**Figure:**
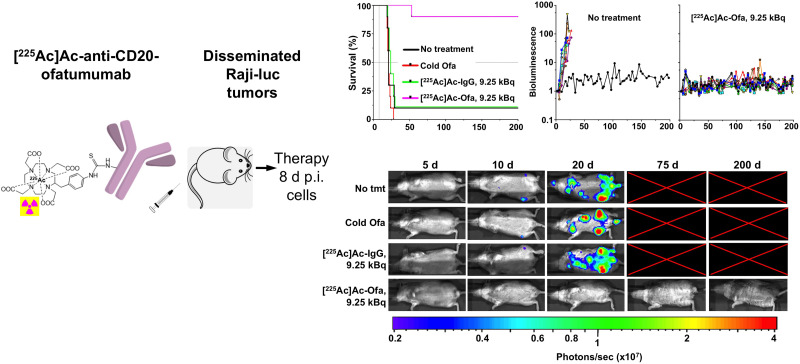

Non-Hodgkin lymphoma (NHL) will be diagnosed in approximately 80,000 patients and cause over 20,000 deaths in the United States in 2023 (1). Although chemotherapy is initially effective, many patients, even with low-grade lymphomas, relapse (2). This has driven development of therapeutic antibodies that target the CD20 protein expressed on the surface of mature B cells and most NHL cells, as most are of B-cell origin. Anti-CD20 immunotherapy has a highly favorable safety profile, significantly improves the outcomes of most patients, and, along with chemotherapy, is now part of the standard of care for many cases of NHL.

The chimeric mouse–human monoclonal antibody, rituximab, was the first Food and Drug Administration–approved anti-CD20 therapeutic, with others subsequently developed for improved biologic and pharmacologic properties, including fully human ofatumumab (3). Ofatumumab binds CD20 with high affinity, allowing targeting of cells with low CD20 expression, including those with resistance to rituximab (4). As a type I anti-CD20 antibody, ofatumumab is effectively internalized (5), which benefits imaging and therapy using residualizing radiometals, such as ^89^Zr and ^225^Ac.

Lymphoma is highly susceptible to ionizing radiation; however, external-beam irradiation is used sparingly in the disseminated setting. To overcome the limitations of external-beam radiotherapy, systemically administered β-particle–emitting radioimmunotherapies to anti-CD20 have been translated into 2 Food and Drug Administration–approved drugs: murine ^131^I-tositumomab (Bexxar; GlaxoSmithKline) and ^90^Y-ibrutumomab (Zevalin; Acrotech Biopharma LLC) (6). Although studies show long-term safety and effectiveness (7), Bexxar has been discontinued commercially in the United States, and Zevalin is used infrequently (8*,*^9^).

^225^Ac has gained increased use as a therapeutic radionuclide (10), with its 10-d half-life matching well the pharmacokinetics of intact antibodies. In its decay pathway, ^225^Ac yields 4 net α-particles with high linear energy transfer and short pathlengths, providing effective radiotoxicity to targeted tumor cells while relatively sparing nontargeted cells.

We previously demonstrated that [^89^Zr]Zr-ofatumumab has excellent uptake into human lymphoma xenografts and enables in vivo localization using PET as well as, or better than, [^89^Zr]Zr-rituximab (11). Here, we prepare [^225^Ac]Ac-ofatumumab and demonstrate potent cytotoxicity to CD20-expressing cells in vitro and in vivo. In therapeutic studies using an aggressive, disseminated murine model of human lymphoma, [^225^Ac]Ac-ofatumumab shows excellent, often curative, efficacy.

## MATERIALS AND METHODS

### Reagents and Cell Lines

Chemicals and reagents are listed in Supplemental Table 1 (supplemental materials are available at http://jnm.snmjournals.org). Water (MilliQ Integral 5 system; Millipore) was treated with a 50 g/L concentration of Chelex 100 (Bio-Rad Laboratories, Inc.). Raji and Raji-Luc cells were cultured in RPMI medium with 10% fetal bovine serum.

### DOTA Conjugation, ^225^Ac Chelation, Radiochemical Yield, Purity, and Mass Spectrometry

DOTA was dissolved in H_2_O and conjugated to antibodies as previously described (11). For ^225^Ac chelation, 1.85 MBq of ^225^Ac in 0.2 M HCl was added to 2 M tetraethyl-ammonium-acetate to obtain pH 6.0. One hundred micrograms of DOTA antibody prepared at an 8:1 DOTA-to-antibody molar ratio were added, and the reaction was brought to 150 μL with 20 mM sodium acetate, 150 mM NaCl (pH 7.0), and 15 μL of a 10 mg/mL solution of sodium ascorbate. After 4 h at 37°C, diethylenetriaminepentaacetic acid (pH 7.0) was added to 5 mM final concentration for 10 min followed by size-exclusion column purification into saline with sodium ascorbate added to 10 μg/mL. All quantifications were at secular equilibrium, using a Capintec CRC 55tW dose calibrator and a Beckman 8000 γ-counter with a 250- to 480-keV energy window or by scanning of thin-layer chromatography strips. Fast protein liquid chromatography, thin-layer chromatography, and mass spectrometry were performed as previously described (12).

### Immunoreactivity, in Vitro Stability, and Cell Killing

Immunoreactivity was assayed by Raji-cell binding as previously described (11), without or with cold ofatumumab blocking. To assay stability, [^225^Ac]Ac-ofatumumab or ^225^Ac was added to human serum, incubated at 37°C, and assayed by thin-layer chromatography as previously described (11).

To assay cell killing, 5 × 10^5^ Raji-luciferase cells in 1 mL of RPMI medium with 10% heat-inactivated fetal bovine serum were added per well, followed by no antibody, native ofatumumab, [^225^Ac]Ac-IgG, or [^225^Ac]Ac-ofatumumab. When used, the antibody mass was 0.1 μg/well. Medium was exchanged after 24 h, and viability assays were performed 48 h later by MTS (3-(4,5-dimethylthiazol-2-yl)-5-(3-carboxymethoxyphenyl)-2-(4-sulfophenyl)-2H-tetrazolium) or bioluminescence imaging as previously described (12).

### Biodistribution in Tumor-Bearing Mice

The Washington University in St. Louis Institutional Animal Care and Use Committee approved the animal studies. Eight- to 10-wk-old R2G2 mice (no. 021; Envigo) were inoculated subcutaneously with 5 × 10^6^ Raji-Luc cells. When palpable tumors were present, 8–11 μg (4.07 kBq) of [^225^Ac]Ac-ofatumumab were administered intravenously and biodistribution analyzed 7 d later. The femur was measured after marrow extraction.

### Therapy and Bioluminescent Imaging

R2G2 mice were injected intravenously with 1 × 10^6^ Raji-Luc cells. In a first study, 8 d later, the mice either were untreated or were treated with ofatumumab or 3.7 or 9.25 kBq/mouse of [^225^Ac]Ac-IgG or [^225^Ac]Ac-ofatumumab. In a second study, 12 or 16 d after cell injection, the mice either were untreated or were treated with 9.25 kBq of [^225^Ac]Ac-ofatumumab. The administered antibody mass was adjusted to 20 μg/mouse of IgG or ofatumumab. Bioluminescent images were acquired and quantified as previously described (12*,*^13^) and normalized to the first imaging time point. The mice were humanely euthanized if they had hind-limb paralysis (HLP), more than a 20% weight loss, or morbidity or reached the scheduled study termination point.

### Biodistribution and Dosimetry in Wild-Type Mice

Biodistribution and dosimetry studies were performed on 6- to 8-wk-old female C57BL/6N mice (no. 556; Charles River) injected intravenously with 3 μg (∼3.7 kBq) of [^225^Ac]Ac-ofatumumab. At selected time points after injection of [^225^Ac]Ac-ofatumumab, the mice were euthanized and organs γ-counted at secular equilibrium to determine decay-corrected percentage injected activity per gram (%IA/g). Bone (tibia and fibula) was counted after marrow separation.

Integrated time–activity curves from the murine data and the mean absorbed dose (*D*) were calculated according to MIRD methodology (14*,*^15^) using the formula *D = A ×* Δ × ϕ, where *A* is the cumulated activity, Δ is the mean α-particle energy, and ϕ is the absorbed fraction, with extrapolation to infinity, yielding a maximally conservative estimate. α particles were assumed to deposit all their energy locally (ϕ = 1). The trapezoidal rule was used to integrate the time–activity curve of the α-particles emitted from the decay of ^225^Ac and its α-particle–emitting daughters (^221^Fr, ^217^As, ^213^Bi, and ^213^Po) using values from International Commission on Radiological Protection publication 107 (16), with all daughter decays assumed to occur in the same organ as the ^225^Ac decay, yielding a Δ of 4.42^−12^ J/(Bq⋅s) for ^225^Ac and its daughters. These mouse data were extrapolated to the adult female human model using the relative organ mass scaling method. Equilibrium in the decay chain and no translocation during the decay between succeeding disintegrations were assumed. Thus, the same estimated integrated time–activity curve obtained for ^225^Ac was applied to its daughters. The absorbed dose of [^225^Ac]Ac-ofatumumab was summed after applying weighting factors in the 2 possible pathways, 2% for ^209^Ti and 98% for ^213^Po. A relative biological effectiveness of 5 for α-particles was used in the calculation of sieverts.

### Statistical Methods

GraphPad Prism software, version 8.4.3, was used for statistical analyses. A *P* value of less than 0.05 was considered statistically significant, with statistical tests noted in the text or figure legends. Data are shown as mean ± SD.

## RESULTS

### [^225^Ac]Ac-Ofatumumab Synthesis, Radiochemical Yield and Purity, Chelate Number, Immunoreactivity, and Serum Stability

[^225^Ac]Ac-DOTA-ofatumumab was prepared using a 1-step method (17) and characterized (Supplemental Figs. 1A–1G). Radiochemical purity was more than 95%, and radiochemical yields were more than 30%, with more than 70% immunoreactivity and specific activities of 5.25–15.6 MBq/mg. An average of 1, 2, and 5 chelates were attached per antibody using DOTA-to-ofatumumab ratios during conjugation of 2:1, 5:1, and 8:1, respectively. Chelation was stable after 10 d in serum.

### Biodistribution with Subcutaneous Raji-Cell Tumors

We used immunodeficient R2G2 mice (B6;129-*Rag2^tm1Fwa^ II2rg^tm1Rsky^*/DwlHsd) that are proficient in double-strand DNA-break repair. *Prkdc^scid^* mice that lack double-strand DNA repair because of the *scid* mutation are known to show artifactual radiosensitivity to DNA damage (18), such as that induced by α-particles. Compared with *Prkdc^scid^* mice, we expect that the nontargeted (nontumor) cells in R2G2 mice will better reflect the response of nontargeted (nontumor) cells in humans to α-particle transit, as both tumor and nontumor cells are proficient in double-strand DNA-break repair.

The biodistribution of [^225^Ac]Ac-ofatumumab was evaluated 7 d after injection in mice bearing subcutaneous Raji tumors (Supplemental Fig. 1H). The radioimmunotherapeutic showed a long circulatory residence time, consistent with stable chelation. This was confirmed by low liver uptake (7 ± 1 %IA/g), as free ^225^Ac shows high liver uptake (19). Splenic uptake was 31 ± 6 %IA/g, similar to that of [^89^Zr]Zr-rituximab and -ofatumumab (11). Marrow and femur showed 7 ± 3 and 5 ± 0.05 %IA/g, respectively. Tumor targeting was 28 ± 10 %IA/g.

### [^225^Ac]Ac-Ofatumumab–Mediated Cell Killing

**FIGURE 1.**
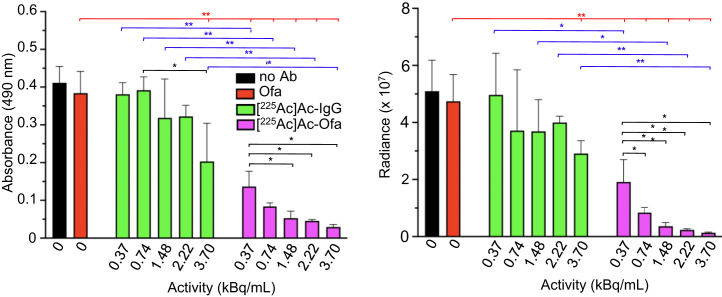
[^225^Ac]Ac-ofatumumab in vitro cytotoxicity to Raji-Luc cells. Cell viability with no treatment control or after exposure to [^225^Ac]Ac-IgG, or -ofatumumab was measured using MTS (left) or bioluminescence (right) assays (*n* = 3). Comparison was by ANOVA (red lines) or Student *t* test (black and blue lines). *P < 0.05. **P < 0.005. Ab = antibody; Ofa = ofatumumab.

To investigate in vitro cytotoxicity, Raji-Luc cells were incubated with medium only, native (cold) ofatumumab, [^225^Ac]Ac-IgG, or [^225^Ac]Ac-ofatumumab for 24 h, followed by medium exchange and, 48 h later, viability assays (Fig. 1). Compared with no antibody, native ofatumumab did not affect viability. A 3.7 kBq/mL dose of [^225^Ac]Ac-IgG showed a modest (∼2-fold) effect, with none at lower doses. [^225^Ac]Ac-ofatumumab yielded significant, dose-dependent reductions in viability compared with cells without antibody or cells exposed to native ofatumumab or [^225^Ac]Ac-IgG.

### Biodistribution and Absorbed Dose of [^225^Ac]Ac-Ofatumumab in C57BL/6N Mice

The biodistribution of [^225^Ac]Ac-ofatumumab was evaluated using non–tumor-bearing, wild-type C57BL/6N mice (Supplemental Table 2). Blood [^225^Ac]Ac-ofatumumab levels were high (43 ± 6 %IA/g) at 4 h after injection and slowly fell to 15 %IA/g at 12 d after injection. Uptake of ^225^Ac-ofatumumab was 5–8 IA/g in bladder, 8–11 %IA/g in kidney, and 6–12 %IA/g in marrow, with a gradual diminution in marrow over time. Liver uptake was 10–15 %IA/g, with perhaps some contribution by dechelated ^225^Ac, which accumulates in the liver (20). Fecal ^225^Ac levels were consistent with [^225^Ac]Ac-ofatumumab or metabolites being excreted via the gastrointestinal route.

[^225^Ac]Ac-ofatumumab human radiation dosimetry estimates were then determined for a human female model (Table 1). As free ^213^Bi in the kidney was not evaluated, this absorbed dose may be somewhat underestimated (21). Extrapolated human radiation dose estimates reveal heart wall (1,919 mSv/MBq) as the organ with the highest predicted dose, followed by liver, spleen, and red marrow at 1,833, 1,803, and 1,620 mSv/MBq, respectively. Marrow is likely the dose-limiting organ. The calculated effective dose equivalent was 1,496 mSv/MBq.

**TABLE 1.** Extrapolated Human Radiation Dose Estimates for [^225^Ac]Ac-Ofatumumab

Organ	Equivalent dose (mSv/MBq)
Adrenals	400
Bladder wall	275
Brain	84
Breast	260
Gallbladder	235
Heart wall	1,919
Intestine, small	247
Intestine, large, lower	260
Intestine, large, upper	251
Kidney	1,187
Liver	1,833
Lung	1,076
Marrow, red	1,620
Muscle	313
Ovaries	943
Pancreas	507
Skin	260
Stomach wall	240
Spleen	1,803
Thymus	638
Thyroid	517
Uterus	1,446
Effective dose	883
Effective dose equivalent	1,496

Mean absorbed doses after injection of [^225^Ac]Ac-ofatumumab into mice, extrapolated to adult human female model.

### Therapeutic Evaluation in a Disseminated Model on Day 8 After Cell Injection

The magnitude, duration, and tumor targeting of the radiopharmaceutical in the biodistribution studies, as well as the in vitro tumoricidal activity, motivated an in vivo lymphoma treatment study. Therapeutic efficacy was evaluated in R2G2 mice with intravenously injected Raji-Luc cells, which become widely disseminated (13*,*^22^*,*^23^). This model recapitulates many features of clinical NHL, as it invades multiple organs, including the hematopoietic compartment. HLP is a frequent cause for censoring.

First, the maximal tolerated dose of [^225^Ac]Ac-ofatumumab in naïve R2G2 mice was identified. Eighty days after injection, 3.7, 11.1, 18.5, and 37 kBq/mouse yielded 5 of 5, 4 of 5, 3 of 5, and 0 of 5 survivors, respectively. Thus, for therapy we used single injections of the nonmyeloablative doses of 3.7 kBq/mouse (low dose) and 9.25 kBq/mouse (high dose).

**FIGURE 2.**
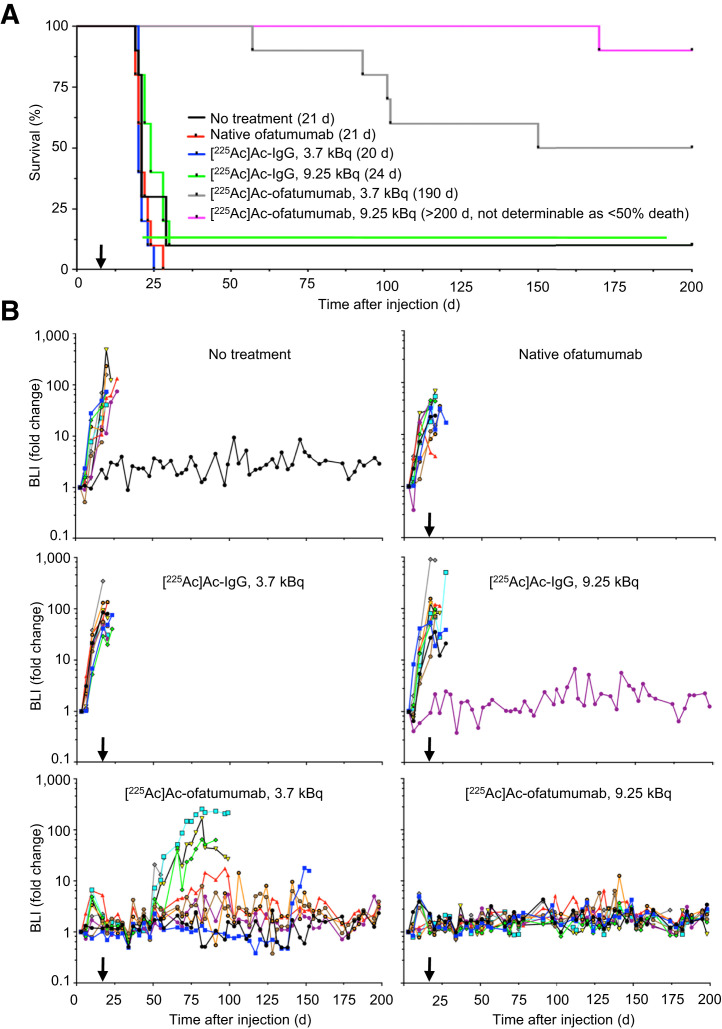
Survival and bioluminescence of mice with disseminated Raji-Luc cells untreated or receiving therapy 8 d after cell injection. (A) Kaplan–Meier graph with median survivals. Low-dose [^225^Ac]Ac-ofatumumab was superior to all other cohorts except high-dose ofatumumab (Mantel-Cox, *P* < 0.05). High-dose [^225^Ac]Ac-ofatumumab was superior to all other cohorts. (B) Bioluminescence. Note elimination of Raji-Luc cells in 9.25 kBq/mouse cohort. Arrow indicates initiation of therapy. *n* = 10/cohort. BLI = bioluminescence.

**FIGURE 3.**
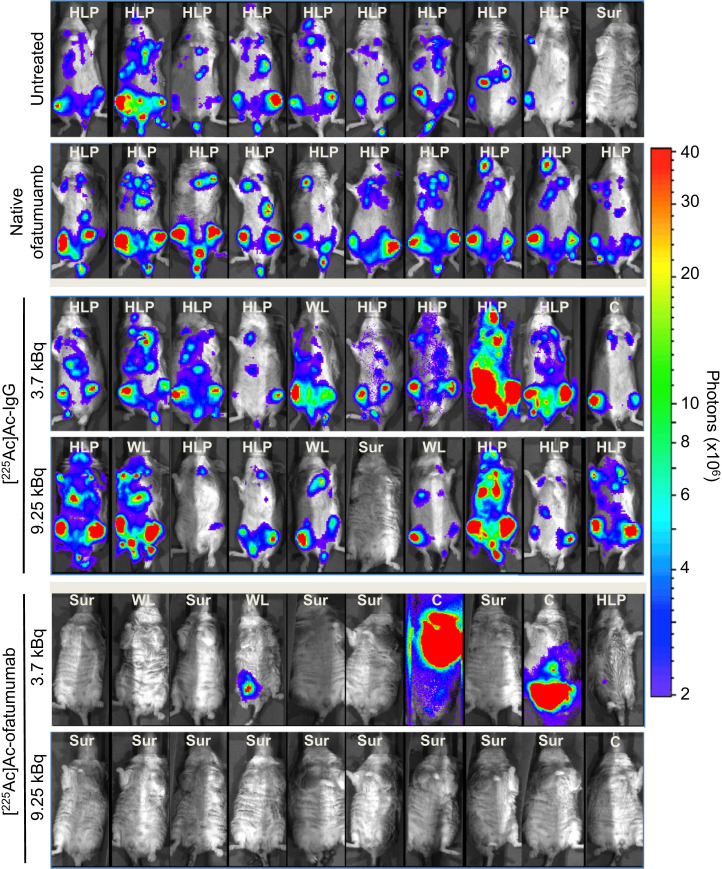
Bioluminescence of mice with disseminated Raji-Luc cells untreated or receiving therapy 8 d after cell injection. Images shown were obtained just before censoring or study termination. Median days of imaging were 20 d for no treatment and ofatumumab; 20 and 23 d for low- and high-dose [^225^Ac]Ac-IgG, respectively; and 152 and 198 d for low- and high-dose [^225^Ac]Ac-ofatumumab, respectively. Note elimination of bioluminescence in mice in latter 2 cohorts. Censoring was for HLP, weight loss, and other causes. C = other causes; SUR = survival; WL = weight loss.

In the first therapy study, 8 d after cell injection the mice were randomized to remain untreated or to be treated with native ofatumumab or low or high doses of [^225^Ac]Ac-IgG or [^225^Ac]Ac-ofatumumab (*n* = 10 mice per cohort). Survival, tumor burden, and weight were monitored for 200 d. In untreated mice, median survival was 21 d, with 9 of 10 mice succumbing before 29 d (Fig. 2A) and all mice showing increasing cancer-cell burden until censoring for HLP (Figs. 2B and 3). The lone surviving mouse never displayed cancer cells, suggesting an unsuccessful injection or engraftment. Native ofatumumab did not extend survival compared with untreated mice, with 10 of 10 succumbing before 29 d (Fig. 2A) and all showing increasing cancer-cell burden until censoring for HLP (Figs. 2B and 3).

Control radiolabeled nonspecific antibody, [^225^Ac]Ac-IgG, at low and high doses yielded median survivals of 20 and 24 d, respectively (Fig. 2A), which did not differ significantly from untreated or native-ofatumumab–treated mice (Mantel–Cox, *P* > 0.05). All mice in the low-dose [^225^Ac]Ac-IgG cohort succumbed before 31 d. Nine were censored for HLP, and 1 perished (Fig. 3). In the high-dose cohort, 6 mice were censored for HLP and 3 for weight loss, with 1 surviving mouse that likely had an unsuccessful injection or engraftment (Figs. 2 and 3). All nonsurviving mice in both cohorts showed continuous cancer-cell growth until they were euthanized (Figs. 2B and 3).

In contrast, low- or high-dose [^225^Ac]Ac-ofatumumab treatment increased median survival to 190 d and more than 200 d (median survival was nondeterminable as there were <50% deaths), respectively (Fig. 2A), superior to survival of untreated or [^225^Ac]Ac-IgG–treated mice (Mantel–Cox, *P* < 0.05). In the low-dose cohort, 5 of 10 mice survived. One mouse was censored for HLP, 2 for weight loss, and 1 for development of a leg tumor; 1 perished (Fig. 3). In the high-dose cohort, 9 of 10 mice survived, with 1 succumbing under anesthesia with no prior morbidity, weight loss, or detectable cancer cells (Figs. 2A and 3), indicating a death unrelated to disease or treatment. The median survival with high-dose [^225^Ac]Ac-ofatumumab was longer than that with low-dose [^225^Ac]Ac-ofatumumab (Mantel–Cox, *P* < 0.05), indicating a dose–response effect.

All mice treated with low- or high-dose [^225^Ac]Ac-ofatumumab that survived had effective cancer-cell suppression with no detectable Raji-Luc cells at study termination (Figs. 2B and 3). The 5 nonsurviving mice in the low-dose cohort showed effective cancer-cell suppression for many days (∼50 d for 4 mice and ∼140 d for 1 mouse), followed by resumption of Raji-Luc cell proliferation, as indicated by increased bioluminescence (Figs. 2B and 3).

**FIGURE 4.**
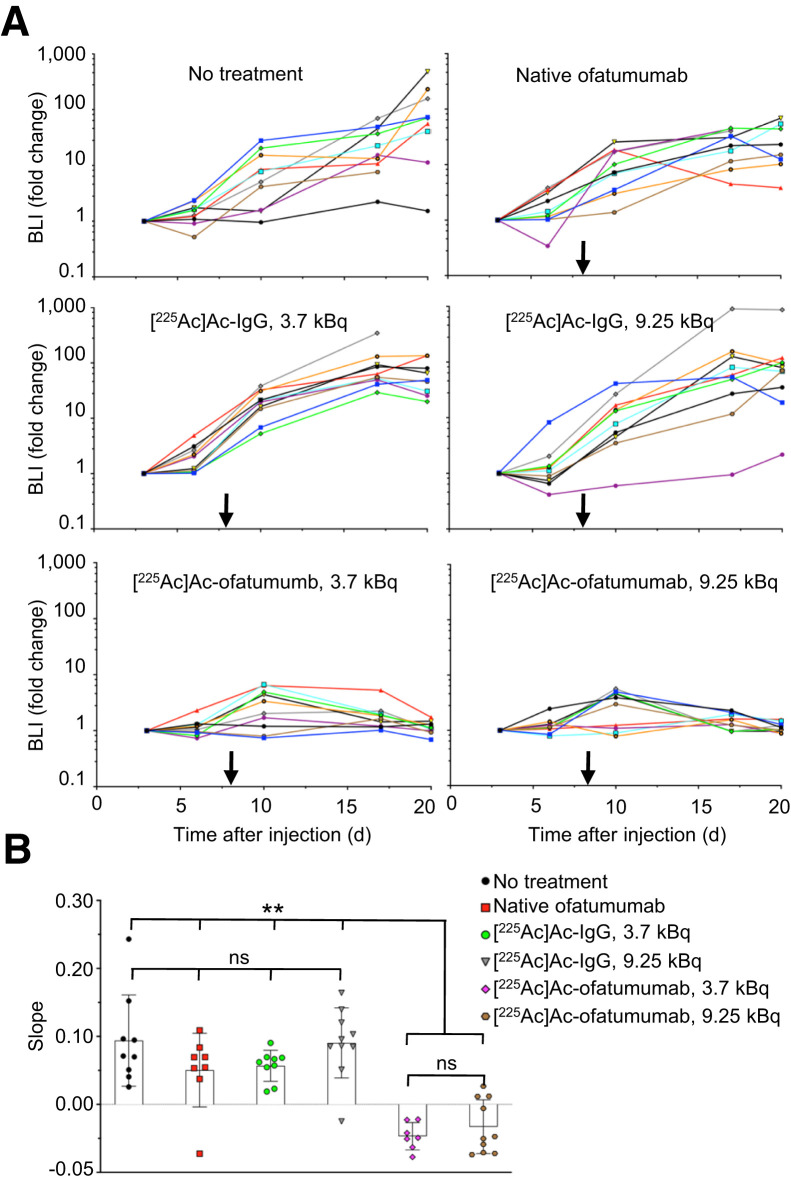
Rapidity of therapeutic effects in mice treated 8 d after cell injection. (A) Bioluminescence of mice 3–20 d after cell injection, showing profound reduction of cancer cell growth rates by [^225^Ac]Ac-ofatumumab. (B) Comparison by ANOVA of bioluminescence from 2 to 10 d after therapy initiation (10–20 d after cell injection). *n* = 7–10/cohort. BLI = bioluminescence; ns = not statistically significant. **P < 0.005.

Because of its 10-d half-life, we wanted to determine how rapidly [^225^Ac]Ac-ofatumumab treatment kills cancer cells. Comparison of bioluminescence from study day 3 to day 20 (5 d before and 12 d after starting therapy) revealed a continued increase in cancer-cell numbers in all cohorts except if treated with [^225^Ac]Ac-ofatumumab (Fig. 4A). To statistically test whether [^225^Ac]Ac-ofatumumab rapidly kills cancer cells in vivo, the log of the bioluminescence values from 10 to 20 d after cell injection (2–12 d after starting therapy) was plotted. Comparison of the slopes of these lines (Fig. 4B) confirmed no effect of native ofatumumab or [^225^Ac]Ac-IgG on cancer-cell proliferation. In contrast, both low- and high-dose [^225^Ac]Ac-ofatumumab significantly reduced these line slopes, indicating rapid killing of cancer cells after initiation of targeted therapy.

### Evaluation of Systemic Toxicity

To investigate systemic toxicity of [^225^Ac]Ac-ofatumumab, animal weights in the therapy study initiated 8 d after cell injection were determined (Supplemental Fig. 2). Not surprisingly, most nonsurviving mice in all cohorts showed clear weight loss before they died or were euthanized for cause, consistent with the increased cancer-cell burden in these mice.

Mice in the low-dose [^225^Ac]Ac-ofatumumab–treated cohort that survived to study termination showed a continuous gradual weight gain (Supplemental Fig. 2B, left). This was consistently slightly lower than the weight gain of control R2G2 mice that were not injected with cells or treated with therapy (Supplemental Fig. 2B), with a significant difference present only after 172 d (Supplemental Fig. 2B, right). Surviving mice in the high-dose [^225^Ac]Ac-ofatumumab cohort showed greater systemic effects, with initial loss of weight having a nadir at 27 d, followed by recovery to initial weight at 52 d and thereafter (Supplemental Fig. 2B).

### Therapeutic Evaluation in a Disseminated Model on Days 12 and 16 After Cell Injection

Next, we asked whether tumor burden at the time of therapy is a relevant parameter in treatment outcome. To answer this question, we delayed treatment from 8 d until either 12 or 16 d after cell injection (untreated mice typically die at 19–21 d after cell injection) to provide a larger pretreatment disseminated disease burden, comparing no treatment with high-dose (9.25 kBq) [^225^Ac]Ac-ofatumumab. To assay tumor-cell burden over time, bioluminescence in untreated mice 8, 12, and 16 d after cell injection was compared (Supplemental Fig. 3) and confirmed a steadily increasing disease burden.

**FIGURE 5.**
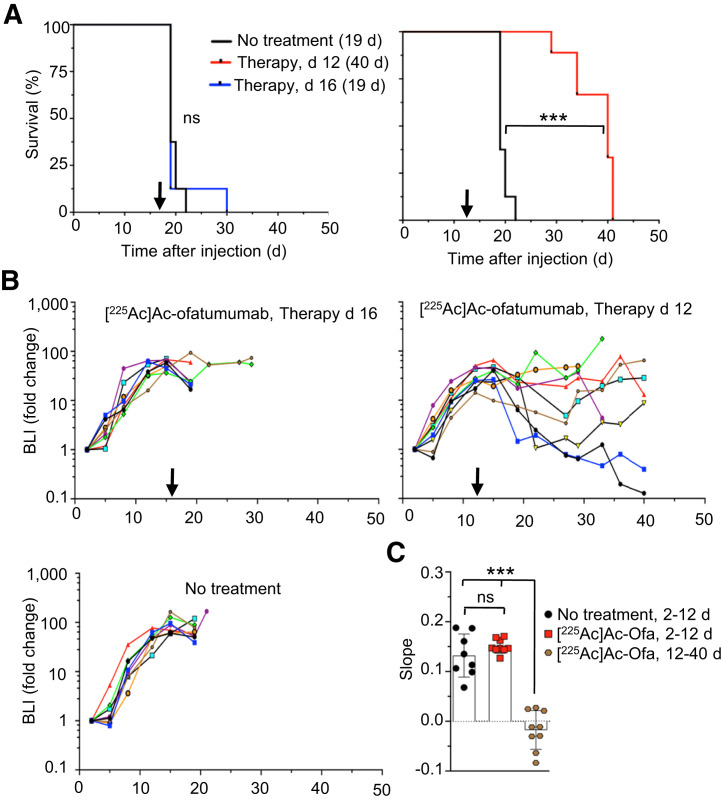
Survival and bioluminescence of mice with larger pretreatment cancer-cell burden. (A) Kaplan–Meier graph of untreated mice vs. treatment started 16 d (left) or 12 d (right) after cell injection, with median survival indicated. Significance was evaluated by Mantel–Cox test. (B) Bioluminescence, showing effect on cancer-cell numbers for cohort treated on day 12 but not day 16. (C) ANOVA comparison of cancer-cell growth of untreated mice or mice treated 12 d after cell injection. *n* = 7–9/cohort. BLI = bioluminescence; ns = not statistically significant. ***P < 0.0001.

When therapy was initiated 16 d after cell injection, [^225^Ac]Ac-ofatumumab provided no survival benefit versus no treatment (Fig. 5A; median survival was 19 d for both cohorts (*P* > 0.05, Mantel–Cox), with a high cancer-cell burden in both cohorts before censoring for HLP (Supplemental Figs. 4A and 4B). Because the time of survival after treatment was very short, the effect on cancer-cell burden past the initiation of treatment was not sufficient for statistical analysis, but any effect appeared minimal (Fig. 5; Supplemental Fig. 4).

In contrast, a single treatment with [^225^Ac]Ac-ofatumumab 12 d after cell injection increased median survival to 40 d (Fig. 5A; Mantel–Cox, *P* < 0.0001), although no mice survived beyond 41 d. This treatment prevented a further increase or yielded a reduction in cancer-cell burden (Figs. 5B and 5C; Supplemental Fig. 4C). In this cohort, 5 mice were censored for HLP and 1 for weight loss; 3 perished (Supplemental Fig. 4C).

## DISCUSSION

Our studies further validate the use of anti-CD20 antibodies for radioimmunotherapy of NHL. We produced [^225^Ac]Ac-ofatumumab with high immunoreactivity, radiochemical yield, and purity and with stable chelation of ^225^Ac in vitro and in vivo. [^225^Ac]Ac-ofatumumab specifically killed CD20-expressing cells in vitro and showed high tumor uptake in vivo. In therapeutic studies using a murine model of disseminated human lymphoma, a single [^225^Ac]Ac-ofatumumab treatment showed excellent efficacy, with curative ability except for very advanced disease.

We prepared [^225^Ac]Ac-ofatumumab using a mild 1-step procedure in which ^225^Ac is directly chelated to DOTA-conjugated antibody at 37°C (17). Other investigators also have used this approach (24–26). ^225^Ac was stably chelated, and uptake of [^225^Ac]Ac-ofatumumab uptake by CD20-expressing subcutaneous tumors (28 %IA/g) was similar to the 33 %IA/g of [^89^Zr]Zr-DFO-ofatumumab (11). The approximately 5 MBq/mg specific activity obtained for [^225^A]Ac-ofatumumab was sufficient for preclinical use and should enable scaling up for clinical use.

The major finding of the current study was the high therapeutic potency of [^225^Ac]Ac-ofatumumab in mice with an aggressive murine model of disseminated human lymphoma, similar to micrometastatic disease in humans. When administered 8 d after cell injection, control unlabeled ofatumumab and low (3.7 kBq/mouse) or high (9.25 kBq/mouse) doses of nontargeted [^225^Ac]Ac-IgG did not improve median survival compared with untreated mice or inhibit cancer-cell growth. In contrast, both low and high doses of [^225^Ac]Ac-ofatumumab rapidly and significantly inhibited cancer-cell growth and increased median survivals to 190 and more than 200 d, respectively. Half the mice in the low-dose group survived, and none of the animals in the high-dose group had disease- or treatment-related mortality. All mice in these 2 cohorts that survived until study termination showed apparent complete elimination of cancer cells, as no cancer cells were detected at study completion on day 200. Also, we tested the potential for systemic anticancer effects in disease settings of further progression. Twelve days after cell injection, high-dose [^225^Ac]Ac-ofatumumab killed cancer cells and significantly extended median survival to 40 d, showing therapeutic benefit even with a high pretreatment burden of cancer cells, although the therapy was not curative. Not surprisingly, there was a limit to therapeutic efficacy; therapy initiated 16 d after cell injection was unable to improve survival. This lack of benefit was likely because approximately 3 d before expected death was not enough time to allow for sufficient targeting and absorbance of the dose of intact radiolabeled antibody, and possibly also because of altered pharmacokinetics due to the high cancer-cell burden (27).

Dosimetry of organs from [^225^Ac]Ac-ofatumumab–injected C57BL/6N mice extrapolated to an adult female human model suggests that marrow will be the dose-limiting organ, as is common with unsealed, intact antibody-based radiotherapeutics, including Bexxar and Zevalin (6). It is worth noting that uptake in nontumor target organs can often be significantly reduced by predosing with unlabeled antibody, which improves lesion–to–background-organ ratios, as used in the Zevalin and Bexxar therapeutic regimens and in other work (28). Fully human ofatumumab is unlikely to induce an immune response, increasing the potential for fractionated dosing of [^225^Ac]Ac-ofatumumab to ameliorate myelotoxicity. In addition, autologous stem-cell transplantation after radioimmunotherapy, as used in some Zevalin and Bexxar protocols, may be useful. Development of a theranostic partner, such as [^89^Zr]Zr-ofatumumab (11), for personalized image-based dosimetry is also of interest. Of course, murine models of toxicity and therapeutic benefit are useful—but imperfect—indicators for treatment of human patients, because of multiple differences including short circulation times in mice and dose–volume geometric considerations. Determination of toxicity–therapeutic trade-offs will require careful evaluation in human trials.

Notably, low-dose [^225^Ac]Ac-ofatumumab treatment 8 d after cell injection was curative in half the mice, which displayed weight gains similar to those of control naïve mice without tumor cells or therapy. The 5 nonsurviving mice in this cohort showed effective repression of cancer-cell growth for approximately 50–140 d and gains of weight over this time of remission. Thus, fractionated cycles of this low-dose therapy may induce cancer-cell elimination, as well as curative efficacy in even more mice while maintaining reduced toxicity. Mice treated with high-dose [^225^Ac]Ac-ofatumumab showed reduced weight gains compared with naïve mice, indicating some systemic toxicity. Some of this may result from kidney distribution of the ^225^Ac daughter radionuclide, ^213^Bi (29), although this possibility remains to be confirmed. However, if so, there are approaches that may ameliorate such toxicity (30*,*^31^).

We recently tested the therapeutic efficacy of β-particle–emitting [^177^]Lu-ofatumumab using this murine disseminated Raji-Luc lymphoma model (12). When therapy was started 4 d after cell injection, 8.51 MBq of [^177^Lu]Lu-ofatumumab showed remarkable therapeutic efficacy, with apparent complete elimination of tumor cells and no disease-related deaths. Our subsequent work (Mark S. Longtine, unpublished data, August 2020), however, indicates that when therapy was initiated 8 d after cell injection, [^177^Lu]Lu-ofatumumab was ineffective, indicating potential superiority of [^225^Ac]Ac-ofatumumab under this condition. Similar observations were noted previously using a murine disseminated multiple-myeloma model, comparing [^177^Lu]Lu- and [^225^Ac]Ac-daratumumab (25).

We recently reported the therapeutic efficacy of rituximab (an internalizing anti-CD20 antibody, like ofatumumab) radiolabeled with ^213^Bi (half-life [t½], 45.6 min; 1 net α-particle), applying a similar disseminated Raji-Luc model using *Prkdc^scid^* mice (23). Cancer-cell killing was effective, and cures were common when the single-dose treatment was started 4 d after injection of 1 × 10^6^ Raji-Luc cells. However, single-dose [^213^Bi]Bi-rituximab treatment was much less effective when initiated 7 d after cell injection, being markedly less effective than single-dose treatment with [^225^Ac]Ac-ofatumumab initiated 8 d after cell injection as found in the current study, although in a somewhat different animal system. These results may in part relate to the decay rates of ^213^Bi versus ^225^Ac and the localization times for intact antibodies to tumor. However, if targets are readily accessible, ^213^Bi can deliver a high dose rate, but if there exist groups of cells, or even solid tumors, with reduced antibody accessibility, sufficient penetration or dose deposition may not occur before radioactive decay, rendering the therapy less effective and yielding superior results with the longer-lived ^225^Ac. In this view, for antibody-mediated radioimmunotherapy for larger tumors, ^225^Ac may be a more appropriate choice for α-particle–mediated therapy. Further evaluation of the comparative therapeutic and off-target effects of ^213^Bi- versus ^225^Ac-labeling for radioimmunotherapy with intact antibodies is of interest.

Other investigators have studied α-emitters for radioimmunotherapy of NHL using preclinical models. Park et al. (32) found therapeutic benefit using a pretargeted approach with a ^213^Bi-labeled anti-CD20 1F5(ScFv)_4_SA molecule. Similarly, [^211^At]At-1F5 (^211^At t½, 7.2 h; 1 net α-particle) was 80% curative 6 d after intravenous cell injection with supporting stem cell transplantation but only poorly effective on subcutaneous tumors (33), perhaps limited by radioactive decay before tumor penetration. [^212^Pb]Pb-rituximab (^212^Pb t½, 10.6 h; 1 net α-particle) in a disseminated model improved survival times with therapy initiated at both low- and high-tumor burdens (34). [^227^Th]Th-rituximab (^227^Th t½, 18.7 d; 5 net α-particles) was often curative of small subcutaneous tumors (35) and was superior to [^90^Y]Y-ibritumomab-tiuxetan, although targeting of the ^223^Ra daughter to bone (36) adds complexity. Similarly, treatment with [^149^Tb]Tb-rituximab (^149^Tb t½, 4.2 h, 1 α-particle) shortly after intravenous injection of Daudi cells significantly increased survival (37). Our excellent results with ^225^Ac labeling likely reflect a very good match between antibody-specific targeting times to tumor and the half-life of this α-particle emitter. In conclusion, a single dose of [^225^Ac]Ac-anti-CD20 ofatumumab showed excellent therapeutic efficacy in a murine model of human disseminated lymphoma and was often curative. Fractionated dosing may improve efficacy. With increasing use of targeted radiotherapies, a renewed application of radioimmunotherapy targeting CD20 for NHL seems warranted, given the exceptional therapeutic efficacy of [^225^Ac]Ac-anti-CD20 ofatumumab and remaining unmet clinical needs in this disease.

## CONCLUSION

[^225^Ac]Ac-ofatumumab shows good in vitro characteristics and effectively targets CD20-expressing tumor xenografts. [^225^Ac]Ac-ofatumumab showed curative efficacy in a murine model of disseminated human lymphoma.

## DISCLOSURE

Richard Wahl is on the scientific advisory board of Clarity Pharmaceuticals, Voximetry, and Seno Medical; has stock in Clarity Pharmaceuticals and stock options in Voximetry; receives honoraria from Bristol Myers Squibb, Actinium Pharmaceuticals, Jubilant Draximage, Siemens, Abderra, and ITM; and receives research support from Actinium Pharmaceuticals, BMS, Bayer, Siemens, and White Rabbit AI. Diane Abou and Daniel Thorek have an advisory board role for, and own stock in, Diaprost AB and Pharma15. This study was supported in part by the Radiological Society of North America (RR1646 to Mark Hoegger), National Institutes of Health (T32EB021955 to Richard Wahl, and R01CA240711, R01CA229893, and R01CA201035 to Daniel Thorek), the Children’s Discovery Institute (MC-II-2021-961 to Diane Abou), the National Institute of General Medical Sciences (5P41GM103422 to the Washington University Biomedical Mass Spectrometry Resource). This work was performed with support of the Washington University in St. Louis Siteman Cancer Center Small Animal Imaging Core. No other potential conflict of interest relevant to this article was reported.

## Notes

KEY POINTS **QUESTION:** Can ^225^Ac-labeled ofatumumab be produced and effectively treat human NHL in a murine model using disseminated xenograft cells? **PERTINENT FINDINGS:** [^225^Ac]Ac-ofatumumab showed stable chelation and was cytotoxic in vitro and in vivo. [^225^Ac]Ac-ofatumumab most commonly showed dose-dependent curative therapeutic efficacy in a murine model of disseminated human lymphoma. **IMPLICATIONS FOR PATIENT CARE:** [^225^Ac]Ac-ofatumumab has excellent potential for translation into the clinic for targeted radioimmunotherapy of patients with NHL.
